# Influence of post-annealing on the off current of MoS_2_ field-effect transistors

**DOI:** 10.1186/s11671-015-0773-y

**Published:** 2015-02-11

**Authors:** Seok Daniel Namgung, Suk Yang, Kyung Park, Ah-Jin Cho, Hojoong Kim, Jang-Yeon Kwon

**Affiliations:** School of Integrated Technology, Yonsei University, Songdo-dong, Incheon, 406-840 Republic of Korea; Yonsei Institute of Convergence Technology, Incheon, 406-840 Republic of Korea

**Keywords:** Molybdenum disulfide, MoS_2_, Field-effect transistors, On/off current ratio, Field-effect mobility

## Abstract

Two-dimensional materials have recently been spotlighted, due to their unique properties in comparison with conventional bulk and thin-film materials. Among those materials, MoS_2_ is one of the promising candidates for the active layer of electronic devices because it shows high electron mobility and pristine band gap. In this paper, we focus on the evolution of the electrical property of the MoS_2_ field-effect transistor (FET) as a function of post-annealing temperature. The results indicate that the off current drastically decreased at 200°C and increased at 400°C while other factors, such as the mobility and threshold voltage, show little variation. We consider that the decreasing off current comes from the rearrangement of the MoS_2_ film and the elimination of the surface residue. Then, the increasing off current was caused by the change of the material's composition and adsorption of H_2_O and O_2_.

**Electronic supplementary material:**

The online version of this article (doi:10.1186/s11671-015-0773-y) contains supplementary material, which is available to authorized users.

## Background

Two-dimensional (2D) materials, such as graphene and transition metal dichalcogenides (MoS_2_, MoSe_2_, WS_2_, etc.), are widely used recently for fabricating next-generation nanoelectronics [1-10]. This is because of the high electron mobility of 2D materials, compared with the original bulk material. Typically, graphene shows over 5,000 cm^2^/Vs of electron mobility [11], and this feature is valuable for applications such as sensors [12] and photovoltaic cells [13]. However, graphene has a fundamental disadvantage for electronic devices, which is the lack of an intrinsic band gap. This has resulted in several reports of insufficient on/off current ratio of field-effect transistors (FETs) [14-17].

Though engineering a band gap of graphene can be an answer for this technical issue, it increases the number of fabrication steps [18,19] and reduces the electron mobility of graphene [20]. As an alternative, MoS_2_ has an intrinsic band gap, which leads to reduced off current. For example, MoS_2_ FETs have in general recorded an on/off current ratio of 10^5^ ~ 10^10^ [21-28], and some MoS_2_ FETs with high-*k* dielectrics have recorded an electron mobility of 200 cm^2^/Vs, which is higher than that of band gap-engineered graphene [21].

Many reports have announced that the annealing process is dispensable for improving the electrical property of various FETs using original IV semiconductors [29], oxide semiconductors [30,31], layered semiconductors [32-34], etc. In the case of 4H-SiC included in the original IV, the annealing process created a passivation layer at the interface, and device parameters were improved, such as the electron mobility and subthreshold swing (SS). In the case of InGaZnO included in oxide semiconductors, the annealing process rearranged defects, and all the device parameters improved, such as *V*_th_, SS, mobility, hysteresis, and the on/off current ratio. For graphene included in a layered material, the annealing process eliminated the resist residue on the surface and increased conductance.

For MoS_2_, a few results have been reported from the viewpoint of the post-annealing process [21,23,26]. One paper showed variation in the optical property, by observing the change of the photoluminescence (PL) peak of single-layer MoS_2_ with respect to post-annealing [35]. Although it did not evaluate the electrical property of FETs, it reported that the annealing process induced structural rearrangement, and this could also affect the electrical properties of MoS_2_. Another paper investigated the influence of vacuum annealing on MoS_2_ FET during measurement of the electrical property [22]. It announced a drastic improvement of electrical performance by annealing, especially in the conductance of the device. However, it focused on the electrical characteristics caused by movement of carriers at elevated temperature, which consequently present the thermally activated characteristics of MoS_2_ FET. Here, we summarize the evolution of the electrical performance of MoS_2_ FET at room temperature, which is the conventional operating temperature, with various post-annealing temperatures.

## Methods

MoS_2_ flakes were prepared using a scotch-tape micromechanical cleavage technique, from bulk MoS_2_ crystal (429ML-AB, SPI Supplies, Inc., West Chester, PA, USA), and were transferred to highly doped silicon substrates covered with 300-nm-thick SiO_2_. Source and drain (S/D) were patterned by photolithography, and 50-nm-thick Ti was deposited by an e-beam evaporator. Then, a conventional lift-off process was accomplished for the patterning of the S/D electrode. The fabricated MoS_2_ FET was annealed in a nitrogen environment for 2 h at various temperatures. The electrical characteristic was measured under atmospheric pressure at room temperature. Furthermore, the thicknesses of the MoS_2_ flakes were measured using atomic force microscopy (AFM; XE-100, Park Systems, Suwon, South Korea).

## Results and discussion

**Figure 1:**
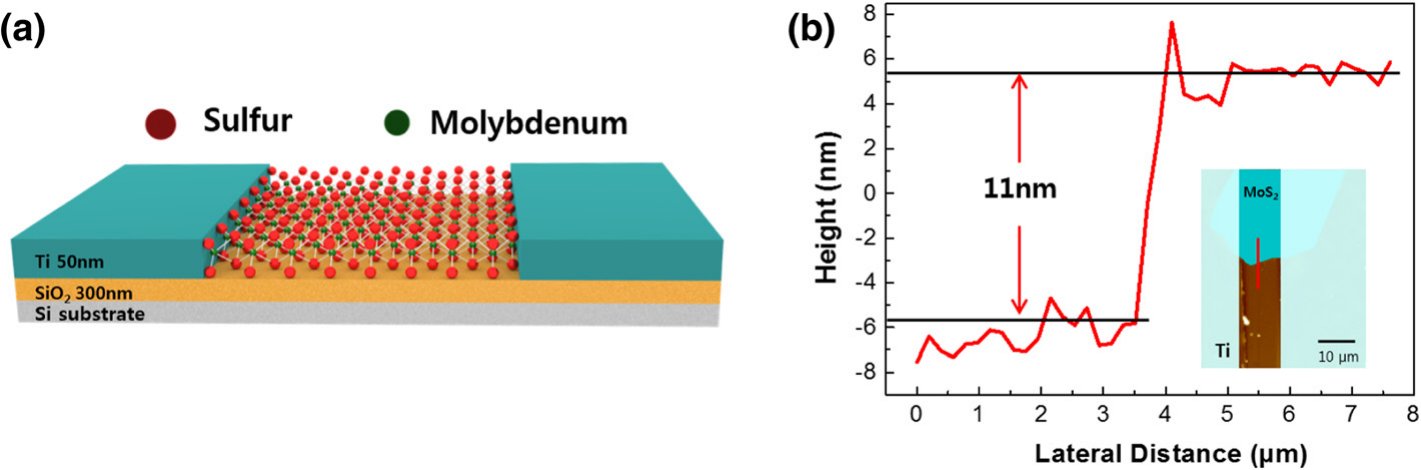
**Schematic representation of MoS**_**2**_ **FET and AFM image. (a)** Schematic representation of MoS_2_ FET with highly doped silicon as the back gate and **(b)** atomic force microscopy (AFM) height profile of multilayer MoS_2_ that has a thickness of 11 nm. The inset is the corresponding AFM image.

Figure 1a is a schematic diagram of the MoS_2_ FET, and Figure 1b is an AFM profile that corresponds to the red line of the MoS_2_ image from the inset. The thickness of the MoS_2_ channel measured by AFM was 11 nm. While there has been controversy over whether using a single-layer MoS_2_ channel is a requirement for getting higher device performance, some papers proved that a multilayer MoS_2_ channel was also able to attain comparable device performance, such as a high electron mobility over 100 cm^2^/Vs and a high on/off current ratio of over 10^6^ [23,36]. Therefore, it is thought that the performance of the multilayer MoS_2_ is sufficient to study the post-annealing effect.

**Figure 2:**
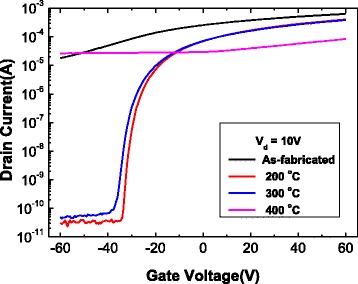
**Transfer curves of the back-gated MoS**_**2**_ **transistor according to the post-annealing temperature.** Transfer curves at room temperature (black), 200°C (red), 300°C (blue), and 400°C (pink) of MoS_2_ FET under various annealing temperatures, at *V*_d_ = 10 V.

Figure 2 shows the representative *I*_d_-*V*_g_ characteristics under constant *V*_d_ = 10 V, with respect to the post-annealing temperature among the many multilayer MoS_2_ FETs shown in Additional file 1: Figure S1. This representative flake has a channel length of 10 μm and a width of 20 μm. This represents the n-type nature of the MoS_2_ channel that makes the accumulation layer of electrons at positive gate biases, and it is observed as increasing the drain current at positive gate biases. Theoretically, the drain current is supposed to be below 10^−9^ A at high negative gate biases, due to a depletion layer; however, a drain current of over 10^−5^ A was observed with various gate biases at room temperature (black line) and at 400°C (pink line). The drain current at the high negative gate biases drastically decreased by approximately 10^6^, compared to that of the device under room temperature, and it seemed that MoS_2_ has a depletion layer at both 200°C (red line) and 300°C (blue line).

**Figure 3:**
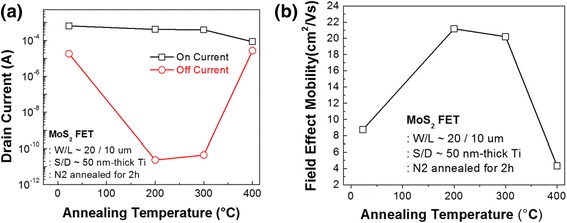
**Trends of on current, off current, and field-effect mobility. (a)** Details of *I*_on_ and *I*_off_ and **(b)** trends of field-effect mobility in terms of annealing temperature.

In Figure 3a, the aforementioned transfer curves are arranged in terms of on and off current, with respect to post-annealing temperatures. The on current was defined as the highest drain current measured at high positive gate biases, and the off current was defined as the lowest drain current recorded at low negative gate biases. Figure 3a shows that the on current consistently decreases as the post-annealing temperature increases, while the off current decreases up to 200°C and increases with further increase of temperature. The lowest value of the off current was observed as approximately 10^−11^ A for the 200°C-annealed device, and this trend is in line with the transfer curve characteristics.

Figure 3b elaborates the field-effect mobility, which increased as the temperature rose and reached a high value of approximately 20.7 cm^2^/Vs at 200°C and 300°C. The field-effect mobility with respect to the post-annealing temperature is also in accordance with the trend of the off current. Table 1 summarizes the details of the FET device performance parameters as annealing temperature.

**Table 1:** **Device performance summary**

**Temperature**	**On/off current ratio**	**On current (A)**	**Off current (A)**	**Field-effect mobility (cm** ^**2**^ **/Vs)**	**Subthreshold swing [V/dec]**
Room temperature	3.5 × 10^01^	6.38 × 10^−04^	1.80 × 10^−05^	8.75	36.20
200°C	1.7 × 10^07^	4.06 × 10^−04^	2.34 × 10^−11^	21.19	0.91
300°C	8.7 × 10^06^	3.87 × 10^−04^	4.43 × 10^−11^	20.21	1.43
400°C	3.2 × 10^00^	8.39 × 10^−05^	2.66 × 10^−05^	4.34	77.51

Exact values of the on/off current ratio, on current, off current, subthreshold swing, and field-effect mobility, at different temperatures.

Under those trends, the status of the device can be categorized into two regions. The first region, here termed region I, is that in which the device performance improves from room temperature to 200°C with decreasing off current and increasing field-effect mobility. The second region (region II) is that in which the device performance degrades from 200°C to 400°C with increasing off current and decreasing field-effect mobility.

In region I, the decrease of off current is thought to be caused by the atomic arrangement of MoS_2_ atoms in local sites due to thermal energy. This kind of internal structural modification ends up with the release of a native point defect at the interface between the insulator and the channel material [30]. The interface properties between the MoS_2_ and SiO_2_ seemed to be improved, in that the subthreshold swing decreased from 36.20 to 0.91 [V/dec], as the post-annealing temperature increased to 200°C.

Also, it is thought that the resist residue included during the fabrication process might be eliminated by the post-annealing process. The photoresist and organic materials from the 3M tape (3M, St. Paul, MN, USA) are one of the plausible candidates to be eliminated, and specifically, elimination of the photoresist residue of the graphene FET was observed with improvement of the device performance during the post-annealing process [33].

In region II, as mentioned, an increase of the off current by 5 or 6 orders was measured.

First, it is thought that such huge increase is caused by the change of the channel material itself. This is supported by the case of oxide semiconductors, such as InGaZnO_4_ where desorption of Zn and O atoms over 700°C annealing and degradation in device performance were observed [30]. Similarly, the results of the X-ray photoelectron spectroscopy (XPS) proved that the S to Mo composition ratio significantly increased after annealing at 400°C in N_2_ (Table 2). Furthermore, time-of-flight secondary ion mass spectroscopy (TOF-SIMS) depth profiles in Additional file 1: Figure S2 show that Mo decreased after annealing at 400°C in N_2_, which correlated with the XPS data.

**Table 2:** **MoS**_**2**_ **composition ratio change based on XPS data**

**Sample condition**	**Atom**	**Atomic %**	**Simplified ratio (let Mo be 1)**
Non-annealed	S	63.14	1.712
	Mo	36.86	1
400°C-annealed	S	69.51	2.280
	Mo	30.49	1

Change of composition ratio between molybdenum and sulfur with respect to post-annealing.

**Figure 4:**
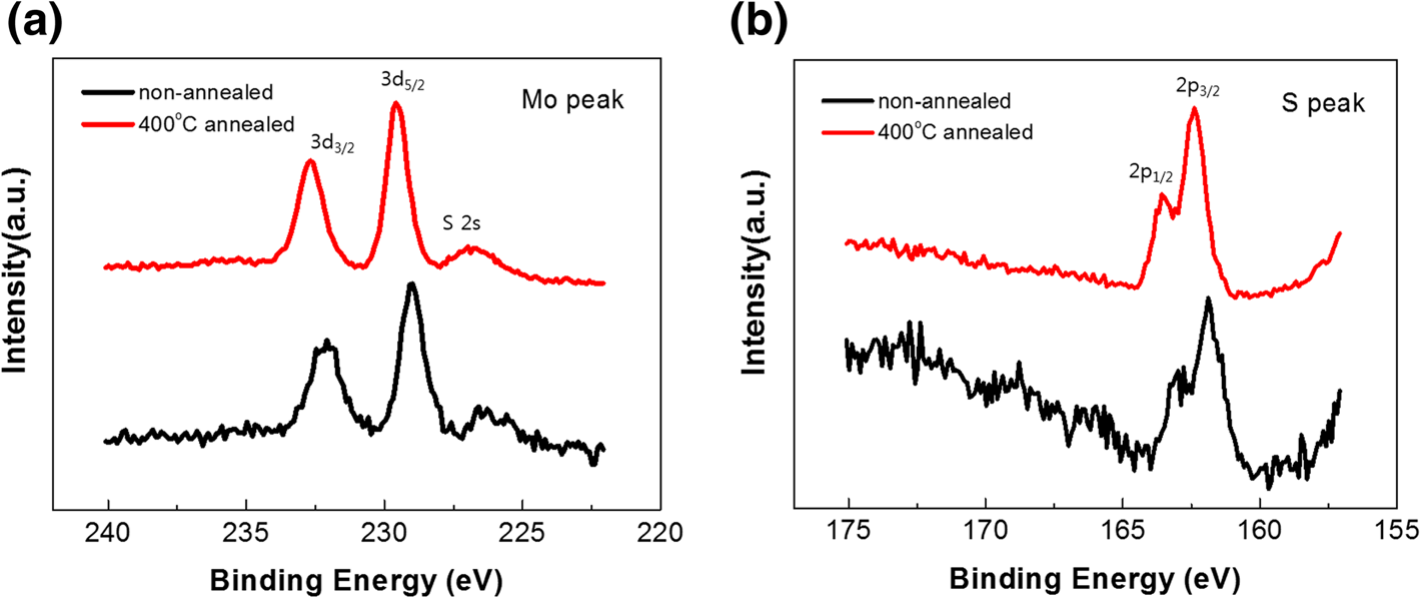
**XPS data of the non-annealed and 400°C-annealed MoS**_**2**_ **. (a)** Molybdenum peak and **(b)** sulfur peak of the non-annealed (black) and 400°C-annealed (red) MoS_2_.

From Figure 4, Mo 3d_5/2_ and S 2p_3/2_ peaks were shifted in a higher energy by 0.6 and 0.5 eV, respectively, after annealing at 400°C. The molybdenum peak shift means that Mo^4+^ (228.98 eV) was changed into Mo^5+^ (230.3 eV) [37], and the S 2p_3/2_ peak shift toward a high binding energy (over 161.88 eV) has been ascribed to polysulfide or thiomolybdate species [38]. That is, one of the strong candidates for explaining the increase of the off current is the phase transformation of MoS_2_ into Mo_2_S_5_ [39] by thermal energy. Furthermore, previous literature [40] provided evidence for this changed form to have high off current in terms of resistivity.

From a different point of view, adsorption of H_2_O and O_2_ on MoS_2_ can also be one of the reasons for the increase of the off current. Under vacuum conditions, the off current actually decreased by average 10^2^ level and this change is elaborated in Additional file 1: Figure S3. Therefore, it is guessed that adsorption was carried out after the high-temperature annealing process for the measurement of electrical characteristics at an atmosphere environment, and it was also supported by the case of graphene [41].

## Conclusions

The evolution of off current for MoS_2_ FET due to annealing temperature was systematically analyzed. As a result, the off current decreased up to 200°C annealing and increased for higher temperature annealing. Plausible explanations for the decrease in off current are the rearrangement of MoS_2_ atoms and the elimination of the surface residue. Possible explanations for the increase in off current are the changes of the material's composition ratio and adsorption of H_2_O and O_2_. This research is meaningful in that the off current was controlled by the post-annealing temperature.

## Additional file

Additional file 1:
**Supplementary figures.** The file contains Supplementary Figures S1 to S3.
