# Supplementary material for: Influence of post-annealing on the off current of MoS2 field-effect transistors
Source: Nanoscale Res Lett. 2015 Feb 11;10:62. doi: 10.1186/s11671-015-0773-y (PMC4385010; doi:10.1186/s11671-015-0773-y)

# Additional File

## Supplementary Figures

**Supplementary Figure S1. Trends of on/off current ratio and off-current at many $MoS_2$ FETs.** (a) Trend of on/off current ratio and (b) Off-current according to post-annealing temperature at many $MoS_2$ FETs

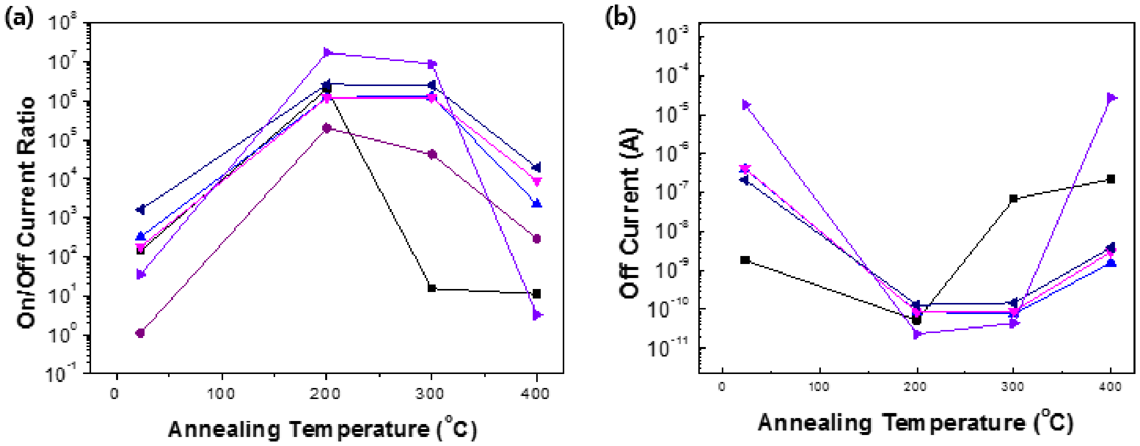

**Supplementary Figure S2. Results of TOF-SIMS depth profile.** TOF-SIMS depth results of (a) non-annealed $MoS_2$ and (b) 400 ℃ $N_2$ annealed $MoS_2$

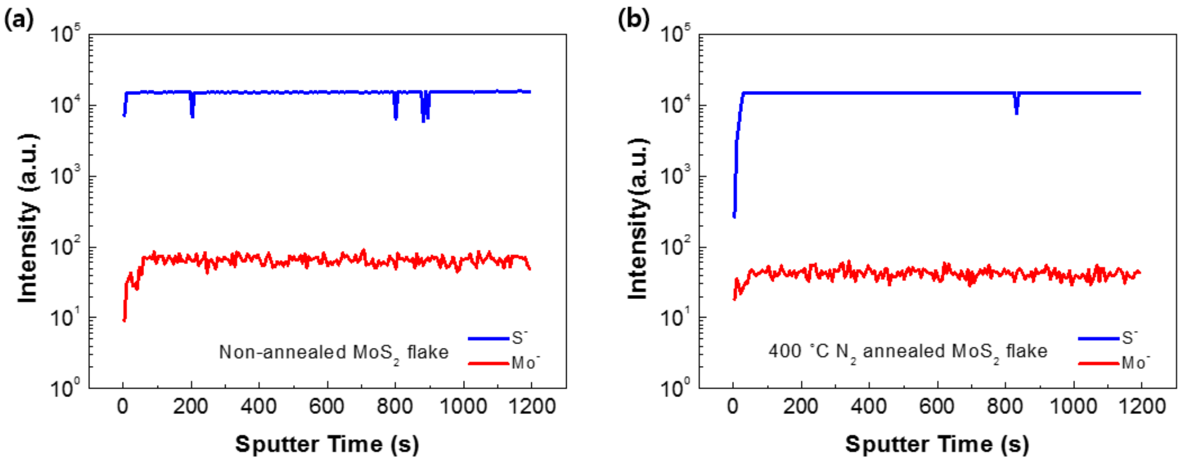


**Supplementary Figure S3. Details of transfer characteristics of each transistor under the atmospheric pressure(dashed) and the vacuum condition(solid).**

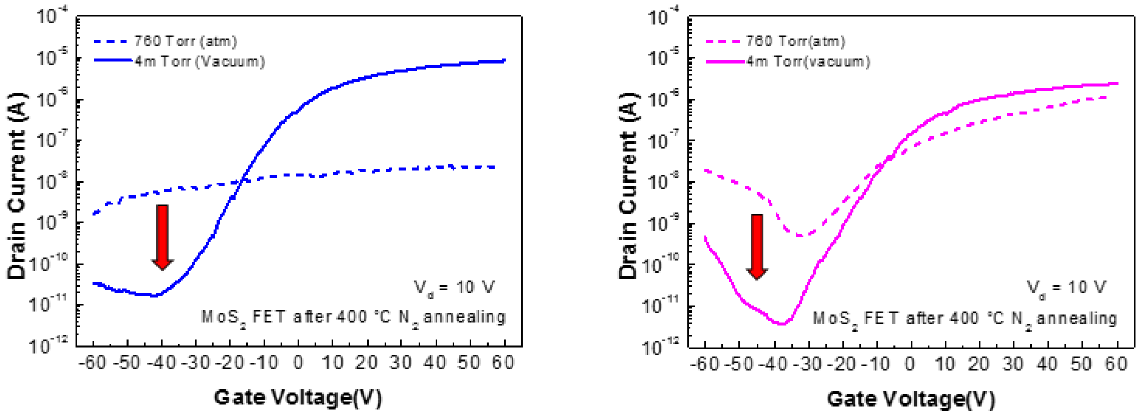

Supplement: Additional file 1: — Supplementary figures. The file contains Supplementary Figures S1 to S3. [file 11671_2015_773_MOESM1_ESM.pdf]
